# Small heat shock protein HSPB5 uses disorder to bind zinc with high affinity

**DOI:** 10.1016/j.jbc.2025.111030

**Published:** 2025-12-09

**Authors:** Maria K. Janowska, Vanesa Racigh, Christoper N. Woods, María Silvina Fornasari, Rachel E. Klevit

**Affiliations:** 1 Department of Biochemistry, University of Washington, Seattle, Washington, United States; 2 Departamento de Ciencia y Tecnología, Universidad Nacional de Quilmes, Bernal, Argentina

**Keywords:** small heat shock proteins, HSPB5, alphaB crystallin, zinc, intrinsic disorder, metalloproteins, chaperone

## Abstract

Zinc is an essential metal that supports diverse cellular functions. Zinc exerts its biological activity through protein binding, serving as catalytic cofactors and structural stabilizers of many enzymes, transcription factors, and ubiquitin E3 ligases, among others. Despite total cellular zinc concentrations reaching hundreds of micromolar, free zinc levels are tightly buffered. Elevated free zinc promotes protein mismetalation and aggregation. While zinc is redox inert, its cysteine (Cys)-based protein ligands are readily oxidized. Oxidative modification of Cys leads to zinc dissociation and a rapid increase in free zinc. With ∼3000 proteins in the human zinc proteome, uncontrolled zinc release could be highly deleterious. Metallothioneins buffer zinc under basal conditions, but their resynthesis following oxidative inactivation occurs on the timescale of hours, raising the question of how free zinc is managed in the interim. Histidine, the second most prevalent zinc-coordinating residue, is resistant to oxidative modification. We characterized zinc binding by the small heat shock protein HSPB5 (αB-crystallin), a Cys-free, histidine-rich protein chaperone that responds to cellular stress and found (1) HSPB5 binds zinc with high affinity and rapid reversibility; (2) zinc binding requires the disordered HSPB5 N-terminal region; (3) zinc binding increases HSPB5 disorder; and (4) prolonged zinc exposure promotes formation of assemblies of oligomers crossbridged by zinc. We propose that HSPB5 has evolved specialized zinc-dependent properties distinct among human small HSPs, enabling it to function not only as a protein chaperone but also as a conditional zinc reservoir under oxidative stress.

Zinc is one of 10 essential metals in humans, supporting processes such as immunity, oxidative stress responses, DNA repair, and aging (1, 2, 3, 4). Unlike redox-active metals, zinc is chemically inert, and its functions arise from protein binding. About 3000 human proteins—roughly 10% of the proteome—require zinc as a cofactor or structural stabilizer (5, 6). Although total intracellular zinc reaches hundreds of micromolar, free zinc is maintained at picomolar–nanomolar levels mainly by metallothioneins, cysteine (Cys)-rich proteins that bind zinc with high affinity (5, 7, 8). Under oxidative stress, Cys thiolates can be oxidized, releasing zinc and elevating free zinc to levels that can drive mismetalation and aggregation (9, 10, 11, 12). Such dyshomeostasis is implicated in disease, including Alzheimer’s plaques and cataractous lenses (13, 14). In proteins, zinc typically coordinates Cys and histidine (His) ligands (5, 6, 15, 16). Because Cys is redox sensitive but His is not, His-rich, Cys-free proteins may serve as conditional zinc buffers when metallothioneins fail.

The small heat shock protein (sHSP) HSPB5 (αB-crystallin) is a compelling candidate for such a role. It is abundant, constitutively and ubiquitously expressed, and acts as a molecular chaperone that protects proteins from aggregation under stress (17, 18, 19, 20, 21). Notably, HSPB5 is enriched in His content with 9 His of 175 residues (5.1%), compared with a 2.6% proteome-wide average (22). Like other sHSPs, it consists of an intrinsically disordered N-terminal region (NTR), a structured α-crystallin domain (ACD), and a disordered C-terminal region (CTR) (23, 24, 25). Interactions among structured ACD dimers, NTRs, and CTRs drive the formation of polydisperse oligomers with ∼12 to 40 subunits, in which the intrinsically disordered regions (IDRs) exist in dynamic equilibrium between solvent-exposed and quasi-ordered states, a property central to sHSP function (21). A typical HSPB5 24-mer therefore contains over 200 His, making HSPB5 a plausible zinc-binding reservoir.

While zinc effects on HSPB5 have been reported (26, 27, 28, 29, 30, 31, 32), direct measurements of binding affinity, stoichiometry, and mechanistic consequences are lacking. We used biochemical and biophysical approaches to define zinc binding to HSPB5. We show that HSPB5 binds zinc with high (nanomolar) affinity, and this requires the disordered NTR. Zinc induces at least two transitions, increases disorder within oligomers, and slows subunit exchange. Prolonged zinc exposure promotes the formation of large species consistent with oligomers crossbridged by zinc. The findings suggest that HSPB5 possesses specialized zinc-dependent properties unique among human sHSPs. We propose that, beyond its established role as a protein chaperone, HSPB5 may function as a conditional zinc reservoir during oxidative stress/zinc distress (33), when Cys-based sites release zinc. This dual role could link proteostasis with zinc buffering and highlights a previously unrecognized dimension of sHSP biology.

## Results

### HSPB5 binds Zn^2+^ with high affinity

**Figure 1:**
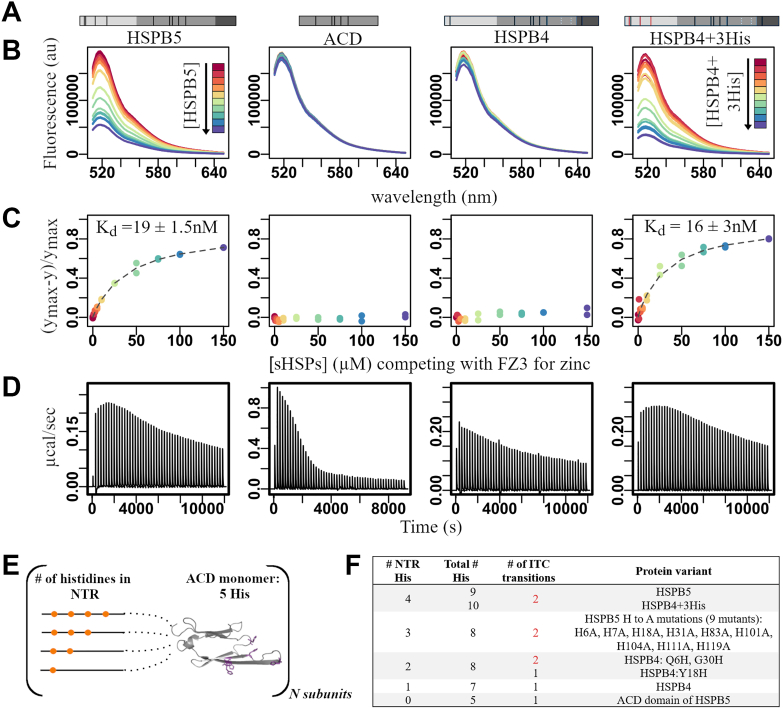
**Zinc binding to HSPB5 depends on disordered NTR histidines (His).***A*–*D* (*left* to *right*), show data for HSPB5, HSPB5 ACD, HSPB4, and HSPB4 with the introduction of three NTR His residues. *A*, scheme showing His positions (*black/red bars*) and removed cysteine residues (*white dotted lines*). Domain boundaries shown in *gray* gradient (NTR, ACD, CTR, and light to dark). *B*, zinc competition assay. Fluorescence emission spectrum of FluoZin-3 (FZ3; 20 μM) in the presence of 1.5 μM zinc with increasing concentrations of sHSPs (0 → 150 μM). Colors correspond to increasing [HSPB5] from *red* to *blue* (*rainbow color scheme*). *C*, fits of assays shown in part *A*. *D*, ITC injection profiles. *E*, scheme showing construct-specific variation in NTR His count. *F*, ITC summary table for all constructs. ACD, α-crystallin domain; CTR, C-terminal region; ITC, isothermal titration calorimetry; NTR, N-terminal region; sHSP, small heat shock protein.

To measure zinc binding, we used a competition assay between a molecule whose fluorescence is zinc dependent and HSPB5 (34). FluoZin-3 (FZ3) binds Zn^2+^ with a *K*_*d*_ of 9.1 nM (35) and fluoresces at ∼520 nm when bound to Zn^2+^. Under conditions of limiting zinc, addition of HSPB5 led to a loss of FZ3 fluorescence, indicating HSPB5 competes for zinc (Fig. 1, *A*–*C*, *first column*). Fitting *F*(520 nm) as a function of [HSPB5] to a simple binding competition equation yielded an apparent *K*_*d*_ of 19 nM. In contrast, no competition for zinc was detected for the ACD dimer, despite containing five His per subunit (Fig. 1, *A*–*C*, *second column*). HSPB4, a closely related sHSP that also forms large oligomers, contains six structurally analogous His to those in HSPB5 and a His in its CTR (Figs. 1*A* and S1). Despite its seven His residues per subunit, HSPB4 did not compete with FZ3 for zinc (Fig. 1, *A*–*C*-, *third column*). Notably, high-affinity zinc binding is recapitulated in an HSPB4 construct to which three NTR His were introduced at positions analogous to HSPB5 positions (HSPB4-3His, Fig. 1, *A*–*C*, *fourth column*). In sum, the results reveal that oligomeric HSPB5 can outcompete FZ3 for zinc, and this high-affinity binding requires the NTR.

### HSPB5 undergoes multiple zinc-dependent transitions that depend on the disordered NTR

We used isothermal titration calorimetry (ITC) to assess zinc binding by HSPB5. Titrations were performed at 50 μM HSPB5, >10^3^-fold above the *K*_*d*_ measured by FZ3, such that stoichiometric binding should yield a linear, monophasic isotherm until saturation. Instead, the observed isotherm was biphasic: an exothermic phase at low zinc ratios (0–1 Zn^2+^/HSPB5) followed by an endothermic phase that saturated at >5 Zn^2+^/HSPB5 (Figs. 1*D*, *first column*, S2, S3, Table S1). In contrast, the HSPB5 ACD dimer produced a simple endothermic isotherm, confirming zinc binding but at much lower affinity than full-length oligomeric HSPB5 (Figs. 1*D*, *second column*, S2, S3, and Table S1). These observations suggest that at least two zinc-dependent processes occur in HSPB5 and implicate the disordered regions in driving the behavior.

Fitting the biphasic isotherm to a two-phase model revealed that both transitions are spontaneous at 25 °C (ΔG < 0). The first transition involved large decreases in both enthalpy (ΔH = −173 kJ/mol) and entropy (TΔS = −146 kJ/mol), whereas the second showed a small enthalpic gain (ΔH = 6 kJ/mol) and a modest entropic penalty (TΔS = −20.5 kJ/mol). Although these values do not permit detailed microscopic interpretation, they establish that zinc binding induces two thermodynamically distinct processes in HSPB5. For comparison, the ACD dimer fits to a single endothermic process (ΔH = 18 kJ/mol; TΔS = −47 kJ/mol), and HSPB4 oligomers also gave a monophasic, endothermic isotherm, confirming zinc binding with lower affinity than HSPB5 (Fig. 1, *D*–*F*, S3, and Table S1).

To probe the contribution of individual residues in HSPB5, each His was replaced with alanine, and isotherms were collected for the resulting oligomers (Figs. 1*F*, S4, and S5). All mutants retained biphasic behavior, showing that no single His is essential for this property (Figs. 1*F*, S4, and S5). We took advantage of the close similarity of HSPB4 to test the role of the NTR His residues. As HSPB4 lacks three of the four His residues in the HSPB5 NTR, single His residues were introduced into a Cys-free version of HSPB4 at positions analogous to HSPB5. Remarkably, His introduction at either position 6 (Q6H) or 31 (G30H) produced HSPB4 variants that display biphasic isotherms, but substitution at position 18 (Y18H) retained the monophasic behavior of wildtype HSPB4 (Figs. 1*F*, and S6). The results indicate that the presence of His at specific NTR positions is sufficient for the biphasic zinc-dependent transitions observed in HSPB5. Ultimately, ITC did not provide a clear stoichiometry of zinc binding to HSPB5 but instead revealed unusual zinc-dependent properties. First, although HSPB5 binds zinc with high affinity, it lacks discrete binding sites composed of defined residue sets. Second, the structured ACD dimer, with 10 His residues, binds zinc with substantially lower affinity than oligomeric HSPB5. Third, ITC detected two spontaneous zinc-dependent transitions. Because the observed heats cannot be directly ascribed to zinc binding under these conditions, we propose they reflect conformational reorganization within or among HSPB5 oligomers that involves the disordered NTR.

### Zinc-induced effects are dependent on dose, time, and temperature and are fully reversible

**Figure 2:**
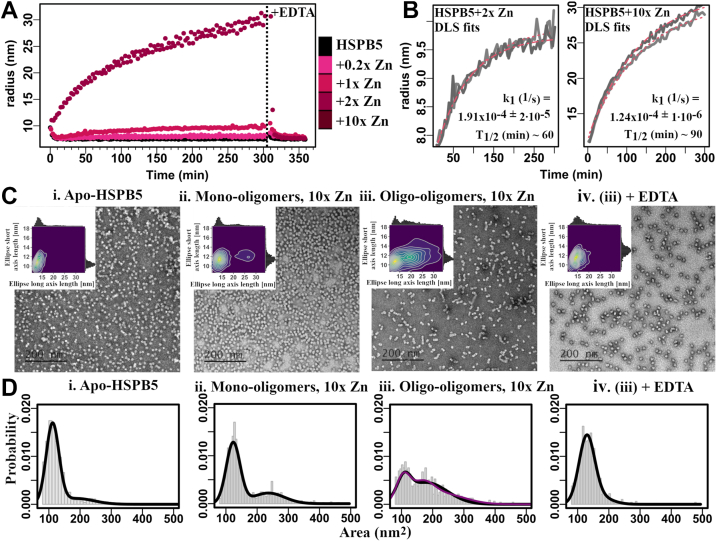
**Zinc-induced increase in HSPB5 size is time dependent and reversible.***A*, average hydrodynamic radius as measured by DLS was monitored over time at 37 °C following addition of 0.2×, 1×, 2×, and 10× zinc (*pink color gradient*). Reversibility was assessed by the addition of EDTA. *B*, fits of DLS time courses for HSPB5 following addition of 2× and 10× zinc. Note the difference in *y*-axis. *C*, conformational rearrangement of HSPB5 in the presence of zinc visualized by negative-stain EM. i. Apo-HSPB5. ii. Mono-oligomers ([−] incubation), 10× Zn, iii. Oligo-oligomers ([+] incubation), 10× Zn, and iv. (iii) + EDTA. *Insets*, EM images were processed, and ellipsoids were fitted into the particles. Plots show 2D density plots of fitted minimum short and long ellipsoid axes. *D*, histograms of area distribution (*gray bars*) with 2- or 3-Gaussian fits (*black* or *purple*, respectively). DLS, dynamic light scattering.

HSPB5 oligomers increase in hydrodynamic radius in the presence of zinc (26). At 37 °C, dynamic light scattering (DLS) measurements showed an average radius of ∼7 nm under low zinc conditions, which remained stable without added zinc but increased over time to ∼8, 10, and 30 nm with 1×, 2×, and 10× zinc, respectively (Fig. 2*A*). Time courses fit to a first-order rate equation yielded condition-dependent rate constants and maximum sizes, with half-times of ∼60 and ∼90 min for 2× and 10× zinc (Fig. 2*B*). These effects occurred at 37 °C but not at 25 °C (Fig. S7). Analytical size-exclusion chromatography confirmed these findings: (1) no elution change with 1× or 2× zinc at 25 °C; (2) zinc-dependent shifts after incubation at 37 °C; and (3) progressive shifts with longer incubation (Fig. S8, *A*–*C*). Together, these results demonstrate that zinc-dependent changes in HSPB5 oligomer size are dependent on the dose, time, and temperature.

The relatively slow time course of zinc-induced effects on HSPB5 raised the question of whether the species formed represent an irreversible soluble aggregate. To test for reversibility, the strong zinc chelator EDTA (*K*_*D*_ ∼10^–16^ M (36)) was added to samples containing high molecular weight HSPB5 species. The average hydrodynamic radius rapidly returned to its initial value following addition of EDTA; the reversal is complete in ∼5 min at 37 °C (Fig. 2*A*). The results indicate that zinc-induced changes in HSPB5 oligomer size are fully reversible and that return to the apo-like state occurs in a matter of minutes at physiological temperature.

DLS indicated formation of large HSPB5 species in the presence of zinc, but because DLS is biased toward large particles, we examined their morphology and prevalence by negative-stain EM (Fig. 2*C*). Zinc-free HSPB5 oligomers appeared symmetric and round, consistent with prior reports (Fig. 2*C*(i)) (19). When applied directly to grids, HSPB5 with 10× zinc looked similar (referred to here as “mono-oligomers”) (Fig. 2*C*(ii)). After 3 h at 37 °C, zinc-containing HSPB5 formed clusters of two to five oligomers in various arrangements (“oligo-oligomers”) (Fig. 2*C*(iii)). Addition of EDTA to these clusters reverted them to zinc-free HSPB5-like species (Fig. 2*C*(iv)), consistent with the DLS results.

Images were analyzed by quantitative particle analysis (37) using two approaches: ellipsoid fitting and surface area calculation for each particle (Table S2). For zinc-free HSPB5, particles appeared spherical, with short and long axis lengths centered around 11 nm, and >80% classified as mono-oligomers, consistent with the known polydisperse size distribution (Fig. 2*C*(i)). Zinc-containing HSPB5 without incubation showed similar axis lengths, but ∼30% of particles exhibited doubled long axes and surface areas, suggestive of two oligomers in contact, whereas ∼70% remained mono-oligomers (Fig. 2*C*(ii)). After incubation with zinc, particles displayed broader distributions in both short and long axes, with greater expansion along the long axis, consistent with end-on-end oligomer associations. In these samples, the mono-oligomer population dropped below 30% (Fig. 2*C*(iii) and Table S2). Finally, the addition of EDTA to zinc-induced clusters restored particles to symmetric shapes comparable to zinc-free HSPB5 (Fig. 2*C*(iv)).

The EM data are congruent with the kinetic parameters obtained from DLS data (Fig. 2*B*). Taken together, they show that HSPB5 undergoes a slow zinc-dependent transition to higher-order oligo-oligomers that dissociate rapidly in the presence of a competitive zinc-binding species. We propose that the oligo-oligomers are composed of two or more oligomers and are stabilized by Zn^2+^ at their interoligomeric interfaces.

### Zinc-dependent conformational and dynamic changes in HSPB5 oligomers

**Figure 3:**
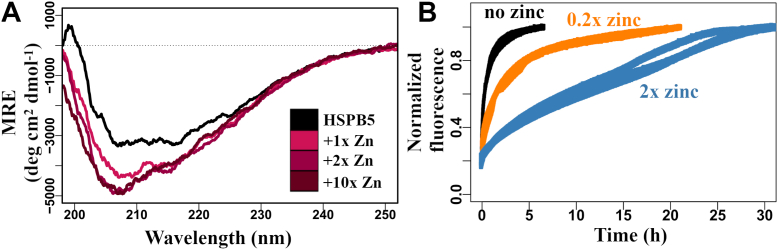
**Zinc induces changes in HSPB5 conformation and dynamics.***A*, secondary structure as monitored by CD at 25 °C. 10 μM HSPB5 was incubated at 37 °C for 3 h in the absence or presence of zinc (0× [*black*], 1× [*pink*], 2× [*dark pink*], and 10× [*maroon*]). *B*, subunit exchange as a function of time at 37 °C in the presence of 0x, 0.2×, and 2×. Data are shown in triplicate.

To assess zinc effects on HSPB5 conformation, far-UV CD spectra were collected for apo-HSPB5 and oligo-oligomer samples formed with 1×, 2×, or 10× zinc. Compared with apo-HSPB5, oligo-oligomers showed a pronounced increase in negative ellipticity with a shift to lower wavelength, consistent with increased disorder (Fig. 3*A*). Time-course CD spectra following zinc addition confirmed that these changes occur over the same period as the slow zinc-dependent alterations observed by DLS and EM. Again, the CD changes were fully reversible with EDTA (Fig. S9). Thus, zinc-driven formation of oligo-oligomeric HSPB5 is accompanied by increased secondary structure disorder.

HSPB5 subunits exchange between oligomers, a property thought to underlie chaperone activity (38), prompting us to ask whether zinc binding affects this process. Subunit exchange kinetics at 37 °C were measured using a fluorescence assay (39) in the absence and presence of Zn^2+^ (Fig. 3*B* and Table S3). Without zinc, the exchange rate was ∼3 × 10^–4^ s^–1^, corresponding to one subunit per hour. Addition of substoichiometric zinc (0.2 mol equivalents per subunit, ∼5 Zn^2+^/oligomer) slowed exchange six fold to ∼7 × 10^–5^ s^–1^ or one subunit every 4 h. Equilibration with 2× zinc (∼48 Zn^2+^/oligomer) produced an even greater 50-fold reduction, yielding a rate of ∼6 × 10^–6^ s^–1^, or one subunit every 50 h. Thus, zinc binding effectively halts subunit exchange, “freezing” subunits within HSPB5 oligomers.

### Structural basis for zinc binding by HSPB5

**Figure 4:**
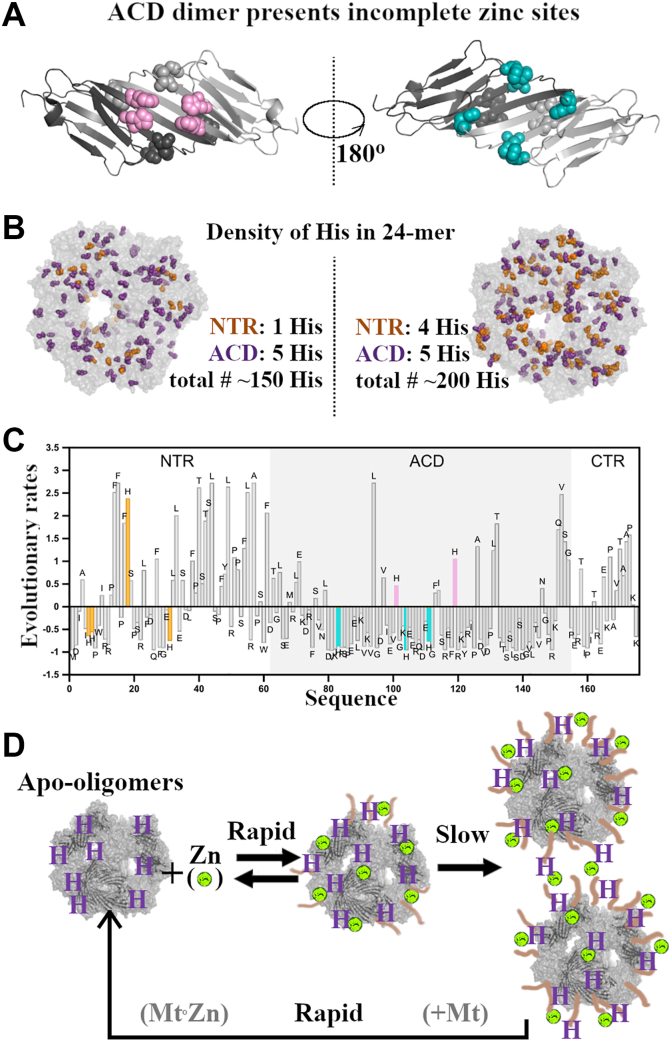
**Structural insights into HSPB5 zinc binding.***A*, HSPB5 ACD dimer (PDB code: 2N0K) showing histidine residues (His): (*Left*) H101 and H119 in *pink*; (*Right*), H83, H104, and H111 in *cyan*. *B*, envelopes illustrating His density in a 24-mer. *Left*, an envelope with six His per oligomer (as in HSPB4’s NTR and ACD). *Right*, an envelope with nine His per oligomer (as in HSPB5). *C*, normalized evolutionary rate profile of human HSPB5. Each bar represents the site-specific evolutionary rate estimated from a multiple sequence alignment of vertebrate orthologs. Rates below zero indicate slower than average evolution, suggesting stronger selective constraints. NTR His are in *yellow*; ACD His are in *pink* and *cyan*, as in *A*. *D*, a possible mechanism of HSPB5-mediated zinc buffering during stress. Zinc (*lime spheres*) can bind to HSPB5 by coordination with His. Prolonged binding leads to conformational rearrangements in HSPB5 and the formation of oligo-oligomers. Zinc can be rapidly released from HSPB5 when canonical zinc-coordinating proteins (*e.g.*, metallothioneins [MTs]) are available. ACD, α-crystallin domain; NTR, N-terminal region; PDB, Protein Data Bank.

HSPB5 and HSPB4 share six structurally analogous His residues, but only HSPB5 displays high-affinity zinc binding and zinc-dependent transitions. What structural features underlie this difference? For reference, we examined zinc-binding proteins with experimentally determined structures that use ≥3 His residues to coordinate zinc. In a representative structure (carbonic anhydrase, Protein Data Bank code: IM1 (40)), His side chains arrange tetrahedrally around Zn^2+^ with interimidazole distances of 3.3 to 3.6 Å (Fig. S10*A*). Each ACD dimer of HSPB4 and HSPB5 contains 10 His residues, with six positioned on one face of the domain and four on the opposite face (Fig. 4*A*). However, in neither case are there three His side chains in close enough proximity to form a Zn^2+^-binding site. There are, however, His pairs in the dimer structure that could form part of a binding site. We conclude that ACD dimers lack preformed zinc-binding sites but could act as partial templates that could be completed by residues contributed by the highly plastic NTR.

A cryo-EM model of HSPB5 (Protein Data Bank code: 2YGD (41)) provides a way to visualize how NTR His residues might participate in zinc binding. Notably, multiple clusters of NTR His are observed: (1) His6/His7 of one chain with His31 of another; (2) His6/His7 with His18 of another; and (3) His31 from three different chains cluster together. Although the reconstruction represents a single oligomeric state within a quasi-ordered, polydisperse, and dynamic ensemble, it suggests that oligomer assembly can juxtapose His residues to form zinc-binding clusters (illustrated in Fig. 4*B*). Functional evidence supports this idea: introducing a single His into HSPB4 at positions equivalent to HSPB5 His6 or His31 confers the biphasic zinc-dependent transitions characteristic of wildtype HSPB5, but introducing His18 does not. These results imply that an NTR containing at least two His residues is required for high-affinity zinc binding and the unusual zinc-dependent behavior. We imagine that pairs of His side chains are presented on an ACD dimer template zinc binding, with sites being completed by many possible combinations of NTR His residues to form a high-affinity tetrahedral coordination of a Zn^2+^ ion. Within oligomers, the NTR His residues may come from different subunits (42), creating an interconnected web and inhibiting the ability of subunits to exchange among oligomers.

To explore whether this property reflects evolutionary pressure, we performed an ortholog-based evolutionary rate analysis of HSPB5 (Figs. 4*C* and S11). The method compares amino acid substitution rates across a multiple sequence alignment, yielding normalized values where negative scores indicate slower-than-average evolution. As expected, most NTR residues evolve rapidly, consistent with intrinsic disorder. Remarkably, however, three of the four HSPB5 NTR His residues (His6, His7, and His31) evolve unusually slowly, a pattern often associated with functional importance in disordered regions (43). Only His18 evolves faster than average. Notably, the functional substitutions above map to the slowly evolving His residue. Although not definitive proof that HSPB5 evolved its His content specifically to support zinc binding, these structural, mutational, and evolutionary observations together suggest that NTR His residues provide the basis for HSPB5’s unique zinc-dependent properties.

## Discussion

Although total cellular zinc concentrations reach the hundreds of micromolar range, nearly all intracellular zinc is protein bound, with free zinc maintained at picomolar–nanomolar levels (7, 8). Most zinc ligands in proteins are Cys residues (15), and with ∼10% of the proteome requiring zinc, the irreversible loss of Cys-based binding sites during oxidative stress could be catastrophic (5). This raises the question of what mechanisms buffer zinc when thiol-based sites are oxidized and zinc is released, a situation known as “zinc distress” (33). Among the small number of Cys-free zinc-binding proteins, HSPB5 stands out as a stress-responsive chaperone with known roles in delaying protein aggregation. This prompted us to ask whether HSPB5 might also function as a zinc sink. We note that properties uncovered here pertaining to zinc could also be relevant to the binding of other transition metals present in a cell. Notably, HSPB5 binds Cu^2+^ with picomolar affinity *via* its ACD, underscoring possible broader metal-buffering potential (26).

The micromolar HSPB5 concentrations in cells exceed free zinc and copper levels, potentially enabling simultaneous coordination of multiple metals. Our results reveal that HSPB5 oligomers, the predominant species in cells, bind zinc with nanomolar affinity and subsequently undergo slow transitions into larger assemblies we term oligo-oligomers. These appear to represent zinc-bound oligomers stabilized by interoligomer crossbridges (30) that likely involve His residues in disordered NTRs. Importantly, zinc-bound oligomers readily revert to their resting state upon chelation with a higher-affinity ligand such as EDTA. Extrapolating to the cellular context, we propose that high constitutive levels of HSPB5 would allow it to rapidly sequester zinc released under oxidative stress and to gradually transition into zinc-storing assemblies. Because metallothionein synthesis takes 24 to 48 h to recover after oxidative inactivation (44), HSPB5 could serve as a temporary buffer to lower free zinc sufficiently to prevent mismetalation and aggregation during this vulnerable period. While zinc-bound oligo-oligomers may have reduced chaperone activity because of their “frozen” subunits, we estimate that each can bind ∼100 zinc ions, a capacity likely sufficient to protect cells from transient zinc overload (Fig. 4*D*).

Beyond revealing a potential zinc storage role, our work highlights the ability of IDRs to coordinate metal ions. In HSPB5, His residues within a disordered region confer high-affinity and high-capacity zinc binding. Importantly, whereas zinc dissociation from most canonical zinc-binding proteins occurs on timescales of days to years under nonstress conditions (16, 45), tightly bound zinc dissociates from HSPB5 in a matter of minutes. Introducing a single additional His into the HSPB4 NTR, which normally contains only one, was sufficient to generate high-affinity binding and zinc-dependent structural rearrangements similar to HSPB5. Remarkably, the context of His matters: substitutions at positions equivalent to H6 or H31 in HSPB5 reproduce the zinc-dependent transitions, whereas substitution at H18 does not (Fig. S6). While the structured ACD also binds zinc, its affinity is much lower. Although the hydrophobic NTR cannot be isolated for direct measurement, results from engineered HSPB4 strongly suggest that zinc binding in HSPB5 arises from the interplay of structured and disordered ligands.

Finally, among the 10 human sHSPs, only HSPB5 is enriched in histidine. Our results suggest that HSPB5’s unusual His content—previously linked to its pH-sensitive activation (20, 21) —also underlies its zinc-binding property, highlighting a broader principle in which IDRs provide adaptive, stress-responsive metal-binding functions. These findings imply an unexpected intersection between proteostasis and metal homeostasis and raise the possibility that HSPB5 has evolved to protect cells not only from protein aggregation but also from zinc dyshomeostasis.

## Experimental procedures

### Mutagenesis and protein preparation

Mutagenesis, expression, and purification of constructs were performed as described previously (46, 47) (see Supporting information for details).

### FZ3 competition assay

Competition assays were performed with excess FZ3 (20 μM; Invitrogen) relative to zinc (1.5 μM ZnCl_2_) and increasing concentrations of HSPB5 in Chelex-treated buffer at 25 °C (34, 35, 48).

### Isothermal titration calorimetry

ITC was performed at 25 °C using 50 μM protein and 2.5 mM zinc. Analysis was performed with MicroCal PEAQ-ITC software (Malvern Panalytical) and NITPIC (49).

### Circular dichroism

CD spectra were collected at 25 °C on 10 μM HSPB5 preincubated with zinc (0×, 1×, 2×, and 10×).

### Dynamic light scattering

DLS time courses were run in duplicate on 10 μM HSPB5 with 0×, 0.2×, 1×, 2×, and 10× zinc (48).

### Subunit exchange assay

Alexa-488–labeled HSPB5 and unlabeled HSPB5 were pre-equilibrated separately with 0×, 0.2×, and 2× zinc. Samples were mixed immediately prior reading on a Horiba Fluorolog-3 (39).

### Electron microscopy

Micrographs of 1 μM HSPB5 in the presence of 0×, 1×, 2×, or 10× zinc stained with uranyl formate (50) were collected on an FEI Morgagni 100 kV TEM.

### Evolutionary analysis

Vertebrate orthologs of human HSPB4 and HSPB5 were retrieved from the National Center for Biotechnology Information Orthologs database https://www.ncbi.nlm.nih.gov/gene (Accessed September 17, 2025). Multiple sequence alignments were generated with Clustal Omega, implemented in UGENE v52.1 (51). Site-specific evolutionary rates were estimated using Rate4Site (52) with default parameters (53, 54).

## Data availability

All data are contained within the article.

## Supporting information

This article contains supporting information (20, 34, 35, 37, 39, 46, 47, 48, 49, 50, 51, 52, 53, 54).

## Conflict of interest

The authors declare that they have no conflicts of interest with the contents of this article.
