# Supplementary material for: Small heat shock protein HSPB5 uses disorder to bind zinc with high affinity
Source: J Biol Chem. 2025 Dec 9;302(1):111030. doi: 10.1016/j.jbc.2025.111030 (PMC12803927; doi:10.1016/j.jbc.2025.111030)
Supplement: Supporting information [file mmc1.pdf]

Title: Small heat shock protein HSPB5 uses disorder to bind zinc with high affinity.

Authors: Maria K. Janowska[1*], Vanesa Racigh[2], Christopher N. Woods[1], María Silvina Fornasari[2], Rachel E. Klevit[1*]

Affiliations:

[1]Department of Biochemistry, University of Washington, Seattle, WA United States

[2]Departamento de Ciencia y Tecnología, Universidad Nacional de Quilmes, Argentina

***Corresponding authors:** mjano@uw.edu, klevit@uw.edu

Materials include:

3 tables

13 figures

Extended Experimental Procedures

Supporting Information

Extended Experimental Procedures

Mutagenesis and protein preparation

All single residue mutants of FL-HSPB5 and HSPB4 were prepared by side-directed mutagenesis using PfuTurbo DNA Polymerase (Agilent). Expression and purification of HSPB4, HSPB5, and His->Ala HSPB5 mutants were performed as described previously (1, 2).

Briefly, HSPB4 or HSPB5 WT and mutants were expressed in Escherichia coli BL21 (DE3) cells (New England BioLabs, NEB). Cells were grown at 37 °C to an OD_{600} of 0.4–0.8, induced with 1 mM IPTG, and incubated overnight at 22 °C. Cells were harvested and resuspended in lysis buffer (20 mM Tris, 100 mM NaCl, 10 mM EDTA, pH 8.0). For purification, cells were lysed using a French press after addition of DNase, RNase, and lysozyme. Polyethylenimine (PEI, 0.1% v/v) was added to the lysate, which was then clarified by centrifugation. Proteins were precipitated with ammonium sulfate (30% w/v), and the resulting pellet was collected by centrifugation. The pellet was resuspended in 20 mM Tris, pH 8.0 (buffer A), and applied to a G25 column equilibrated with buffer A. Desalted protein was pooled, applied to a DEAE column equilibrated with buffer A, and eluted with a sodium chloride gradient. The DEAE eluate was diluted in buffer A and further purified on a HiTrap Q column (Cytiva) using a sodium chloride gradient. The protein was pooled, concentrated, and applied to an SDX200 column (or SDX75 for ACD domain purification) equilibrated with 25 mM MOPS, 150 mM NaCl, pH 7.5. Pure protein was flash-frozen in liquid nitrogen and stored at −80 °C. To ensure lack of binding between buffer and zinc ions, MOPS buffer was used, as it is established that it does not bind zinc. Buffer conditions used: 25 mM MOPS, 150mM NaCl, in pH 7.5 unless specified otherwise. The metal content of the sample was quantified using inductively coupled plasma mass spectrometry (ICP-MS). Analyses were outsourced to the University of Washington Analytical Service Center, and the results are presented in Fig. S12. No significant levels of contaminating or copurifying transition metals were detected. Zinc was added from 20 mM stocks of zinc chloride salts dissolved in water.

Preparation of HSPB5-Zinc oligomers

HSPB5 is highly sensitive to solution conditions. Typically, the response to changes in conditions is fast (as in the case of pH). In comparison, responses of HSPB5 to zinc is a slower process. We monitored zinc-induced transitions by DLS and SEC and found it necessary to incubate HSPB5 with zinc at physiological temperature (37°C) for ~3h; the transitions are not observed at room temperature or on ice (Fig. S7&S8). We used these insights to prepare distinctly different species of HSPB5 for study. To assess HSPB5 conformation, we prepared two zinc-bound states (mono-oligomers and oligo-oligomers) that correspond to the initial conformation upon zinc-binding and the final conformation. To prepare mono-oligomers state, HSPB5 was incubated for 3h without zinc, followed by addition of zinc and storage on ice until the start of an experiment. To prepare oligo-oligomers, HSPB5 was incubated in the presence of zinc for 3h at 37°C. After incubation, samples were placed on ice until an experiment was performed (maximum few hours). We tested that EDTA can strip zinc from oligo-oligomers by monitoring the decrease in size via DLS and learned that the recovery process is temperature-dependent: at 37°C return to apo-state takes ~5 min and at 25°C recovery takes ~40-50 minutes.

FluoZin-3 zinc competition assay

To avoid zinc contamination: buffers (25mM HEPES, 150mM NaCl, pH 7.5) were made with optima water and chelexed overnight. Competition assays included excess FluoZin-3 (20 μM; Invitrogen) relative

to zinc (1.5 µM zinc in the form of $ZnCl_2$) to which increasing amounts of HSPB5 (0, 1, 2, 2.5, 5, 10, 25, 50, 75, 100, 150µM) were added. HSPB5 was incubated for 1h @37°C before the experiment without zinc or FluoZin-3. Assays were performed at 25°C to avoid conformational rearrangement of HSPB5 upon zinc binding. Titration points were performed in duplicate. Two curves are shown for each data point. Experiments were performed on a BMG LABTECH CLARIOstar Plus using low volume black 96 well plate (Corning). Data were fitted assuming the following processes occurring in the sample:

$$Zn + FZ3 \rightarrow Zn - FZ3$$

$$Zn + HSPB5 \rightarrow Zn - HSPB5$$

And solved using quadratic equation for the Kd of sHSPs similarly to Jakob *et al.* (3)

Where:

$$K_{D\ (FZ3)} = \frac{[FZ3]_{free} *\ [Zn]_{free}}{[FZ3]_{bound}}$$

$$K_{D\ (HSPB5)} = \frac{[HSPB5]_{free} *\ [Zn]_{free}}{[HSPB5]_{bound}}$$

And assuming:

$$[FZ3]_{free} = [FZ3]_{total} - [FZ3]_{bound}$$

$$[HSPB5]_{free} = [HSPB5]_{total} - [HSPB5]_{bound}$$

$$[HSPB5]_{bound} = [Zn]_{total} - [HSPB5]_{bound}$$

The resulting quadratic equation had a following form:

$$[FZ3]_{bound} \sim \frac{-b + \sqrt{(b^2 - 4ac)}}{2a}$$

$$a = K_{D\ (FZ3)} - K_{D\ (HSPB5)}$$

$$b = K_{D\ (FZ3)} * [B]_{tot} - K_{D\ (HSPB5)} * [Zn]_{total} + K_{D\ (HSPB5)} * ([FZ3]_{total} - [Zn]_{total})$$

$$c = [FZ3]_{total} * K_{D\ (HSPB5)} * [Zn]_{total}$$

The data were fitted using Levenberf-Marquardt Nonlinear Least-Squares Algorithm with minpack.lm R package (4), using a value of 9.1nM for $K_{d(FZ3)}$ (5).

Isothermal calorimetry

ITC measurements were performed on a Microcal VP-ITC calorimeter (Malvern Panalytical) at 25°C. Proteins in the ITC cell were at 50 µM in 50 mM MOPS, 150mM NaCl. Zinc chloride (2.5 mM) was used

as titrant. Binding curves at pH 7.5 and 6.5 were highly similar, so experiments were performed at pH 6.5 (Fig. S1) to avoid zinc solubility issues. Injection volume was 3uL and injections were in 4-min intervals. Data were analyzed using Microcal PEAQ-ITC analysis software (Malvern Panalytical). The heat of the first titration point was removed from fitting the two sites binding model as per standard ITC analysis practices. Baselines for the plots were calculated using NITPIC software (6).

Circular Dichroism

Circular dichroism spectra were collected on a Jasco J-1500 CD spectrometer with a 1 nm bandwidth on proteins dissolved in 1 mM MOPS, 6 mM NaCl at pH 7.5. For time-course experiments, 2.5 μM HSPB5 was preincubated at 37°C for 1h before 30x zinc addition. After recording the apo-HSPB5 spectrum 75 μM zinc was added and wavelength scans were performed at multiple timepoints for 3h, after which 0.5 mM EDTA was added and another spectrum was recorded. For monitoring zinc dose-dependent changes in the HSPB5 spectrum 10 μM protein was preincubated for 3h at 37°C in the presence or absence of zinc (0x, 1x, 2x, 10x), spectra were recorded at 25°C. All the data shown have a high-tension below 600 V.

Dynamic Light Scattering

Dynamic Light Scattering was performed on a DynaPro Plate Reader III from Wyatt Technology Corporation using Aurora 384-well plate sealed with film to prevent evaporation. Data were analyzed with DYNAMICS 7.8.2 software provided with the instrument. Experiments to monitor the time-course of protein size were performed in duplicate using 10 μM HSPB5 variants in 25 mM MOPS, 150 mM NaCl, pH 7.5 with 0x, 0.2x, 1x, 2x, and 10x zinc addition. Proteins were filtered before experiments using 0.22 μm filters. All samples were incubated for 1h at 37°C before being added to a preheated plate. DLS measurements were started immediately and were performed at 37°C or 25°C (control). EDTA (1 mM) was added to all wells after samples reached a plateau. The increase in size was later analyzed by assuming first order reaction kinetics and fitting to $y = C1 – C2*\exp(-k_1 t)$ using minpack.lm R package (4). C1 represents the maximum radius and k_1 is the apparent rate constant.

Subunit Exchange measured by fluorescence

Alexa Fluor 488 maleimide labeling protocol was described previously (7). 15 μL of 2 μM of S153C HSPB5 labeled with Alexa (81% label efficiency) and 10 μM wild type, unlabeled HSPB5 (135 μL) were preincubated at 37°C for 3h with or without zinc (0, 0.2x, 2x zinc) before starting an experiment. The samples were mixed in a 37°C preheated cuvette just prior to starting an experiment. Experiments were performed on a Horiba Fluorolog-3. Excitation and emission wavelengths for Alexa Fluor 488 maleimide 498/518 nm were used. The increase in size was later analyzed by assuming first order reaction kinetics and fitting to $y = C1 – C2*\exp(-k_1 t)$ (4). All the experiments were performed in triplicate.

Electron microscopy

Micrographs were collected on FEI Morgagni 100 kV TEM equipped with Gatan Orius Camera. 20 μM HSPB5 was incubated with 0 μM, 20 μM, 100 μM, or 200 μM zinc at 37°C for 3h. Additional samples of 20 μM HSPB5 incubated with 200 μM zinc on ice for 3h, with zinc added after incubation at 37°C and 20 μM HSPB5 incubated with 200 μM zinc for 3h then stripped with 1mM EDTA and incubated at 37°C for 30 minutes were prepared. Buffer conditions in 25 mM MOPS, 100 mM NaCl, pH 7.5. Samples were diluted 20x for grid preparation and stained with uranyl formate. Negative-staining for TEM was performed using the single droplet procedure at ambient temperature (8). Samples (3 μl of 2 μM protein variants) were pipetted onto discharged carbon-coated copper grids for 60 s and then were blotted by filter

paper, then stained with drop (5 uL) of Uranyl Formate (0.75%) for 60 s. Stain was blotted with filter paper, and grids were allowed to dry on filter paper and then stored in a container at room temperature.

Images (see Fig. S13) were analyzed by quantitative particle analysis using Fiji (9) in two ways: 1) particles were fit to an ellipsoid and 2) surface area was calculated for each particle (Table S2). The analysis was performed on at least 500 particles from each grid. We compared particle width vs length using the short and long ellipse axis lengths from the ellipsoid fits (insets in Fig. 2C). The surface area analysis of particles was used to estimate relative populations of species under each condition. Histograms of particle surface areas were fitted to a two-Gaussian model (Fig. 2D and Table S2); the oligo-oligomer data were also fit to a three-Gaussian model (Table S2). The area under each curve represents the relative population.

Size exclusion chromatography (SEC)

SEC experiments on a Superose 6 10/300 GL column (GE Life Sciences) were performed on GE AKTA Purifier. Injection volume was 100µM. The zinc concentration effect was accessed by adding different amounts of zinc (0, 1x, 2x) to HSPB5 and incubating for 3h at 37°C. For assessing the importance of the incubation effect on size changes of HSPB5 in presence of zinc 100 µM HSPB5 with 1x zinc was used in 150 mM NaCl, pH 7.5. Different length of incubation of HSPB5 with zinc @ 37°C: 0, 1h, 3h incubation.

Evolutionary analysis

To obtain the site-specific evolutionary rate profiles of human HSPB5 and human HSPB4, vertebrate orthologous sequences were retrieved from the NCBI Orthologs database (https://www.ncbi.nlm.nih.gov/gene), using one representative protein per species. The queries were based on the human genes CRYAB (Gene ID: 1410) and CRYAA (Gene ID: 1409), which encode HSPB5 and HSPB4, respectively. Only orthologous sequences were included to ensure that the dataset reflected the evolutionary history of a single ancestral gene, allowing meaningful estimation of relative evolutionary constraints at each site along the protein sequence. Manual curation was performed to retain only full-length sequences and to exclude those containing undefined residues (e.g., “X”). The final datasets included 369 sequences for HSPB5 and 424 for HSPB4.

Multiple sequence alignments were generated using Clustal Omega, as implemented in the UGENE software (v52.1) (10). Rate4Site program (11) was used with default parameters (JTT as amino acid substitution matrix and neighbor-joining method to reconstruct the phylogenetic tree) (12, 13) to estimate normalized evolutionary rates at each aligned position. Human sequences were used as reference for both analyses (HSPB5: UniProt P02511, HSPB4: UniProt P02489).

Comments about HSPB5 and evolutionary rate estimation:

Residues that evolve at slower rates are typically under positive selective pressure, often reflecting either structural or functional constraints. Given the intrinsically disordered nature of the NTR, the observation that histidine residues within this region exhibit evolutionary rates lower than the sequence average suggests a potential functional relevance.

In contrast, histidines within the ACD are part of a domain that is highly conserved among sHSPs, and residues in the β-strand regions are generally expected to evolve slowly due to their involvement in maintaining domain structure through stabilizing interactions. Histidines located in the ACD have a range of pKa values, suggesting their involvement in different functional aspects (14).

His101 and His119 exhibit scores higher than the sequence average. Both face the exterior surface of the ACD dimer in the 2N0K structure and their imidazole rings are close to each other. Notably, in the MSA, His119 is conserved in only 208 out of 369 sequences (56.4%), whereas His101 is conserved in 345 out of 369 (93.5%). The most frequent substitutions for His119 are asparagine, serine, and glutamine. This suggests that, although in human HSPB5 these two histidines are spatially close and interact within an ACD, their proximity is not strongly conserved across vertebrates.

In contrast, His104, which points toward the inner surface of the ACD, and His83 and His111, located in loop regions, appear to participate in a broader network of interactions via their side chains. His104 is 100% conserved across the MSA and aligns with His100 in HSPB4, which directly coordinates a Zn^{2+} ion in the 3L1G structure. Furthermore, the functional relevance of HIS104 in mediating interface stability and regulating oligomer structure in response to acidosis has been demonstrated (14), suggesting its evolutionary conservation reflects a critical functional role that should be considered when interpreting its score.

Finally, His7, the only His residue within the HSPB5 NTR that is also present in HSPB4, is conserved in 71.3% of the sequences and is replaced by Asn in 27.1%. Notably, while HIS7 is highly conserved in HSPB4 (96.7%), this paralog does not exhibit the high-affinity zinc binding observed in HSPB5. High-affinity zinc binding and zinc-induced transitions are only recovered in HSPB4 when mutations equivalent to HSPB5's His6 and His31 are introduced.

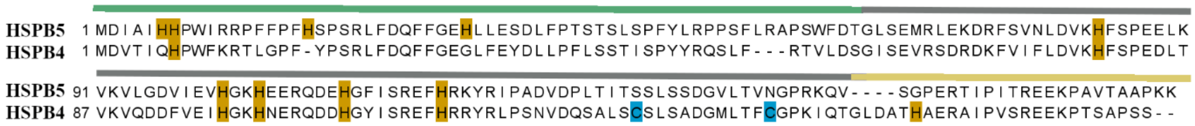

Figure S1 Sequence alignment of HSPB5 and HSPB4. Boundaries of the domains of HSPB4 and HSPB5 are highlighted (green-NTR, grey -ACD, yellow-CTR) above the sequence. Histidine residues are highlighted in orange. Cysteines in HSPB4 are shown in blue. For the ITC experiments the cysteines were substituted with serine.

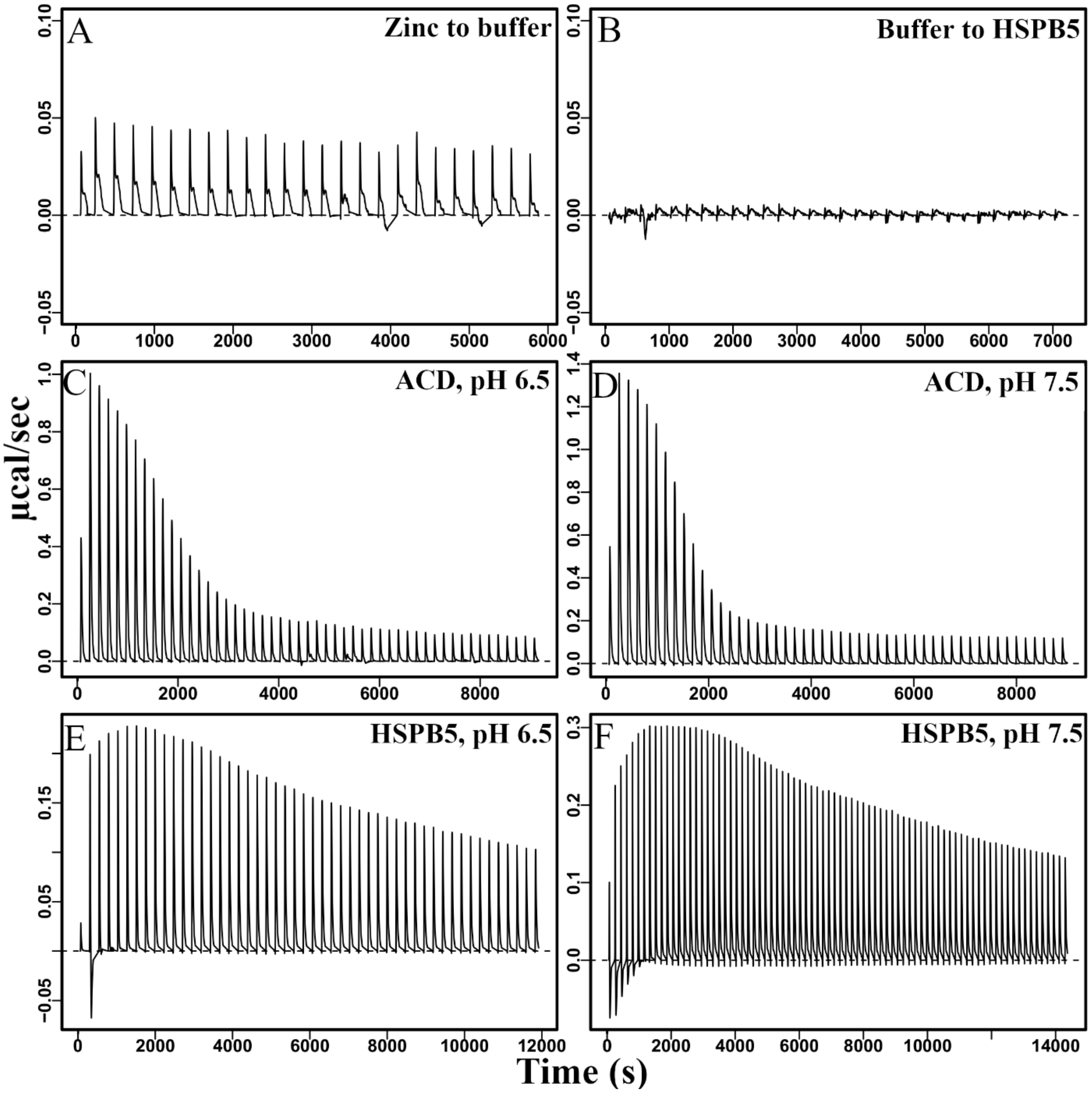

Figure S2 ITC injection profiles. Controls and WT constructs in pH 7.5 and 6.5

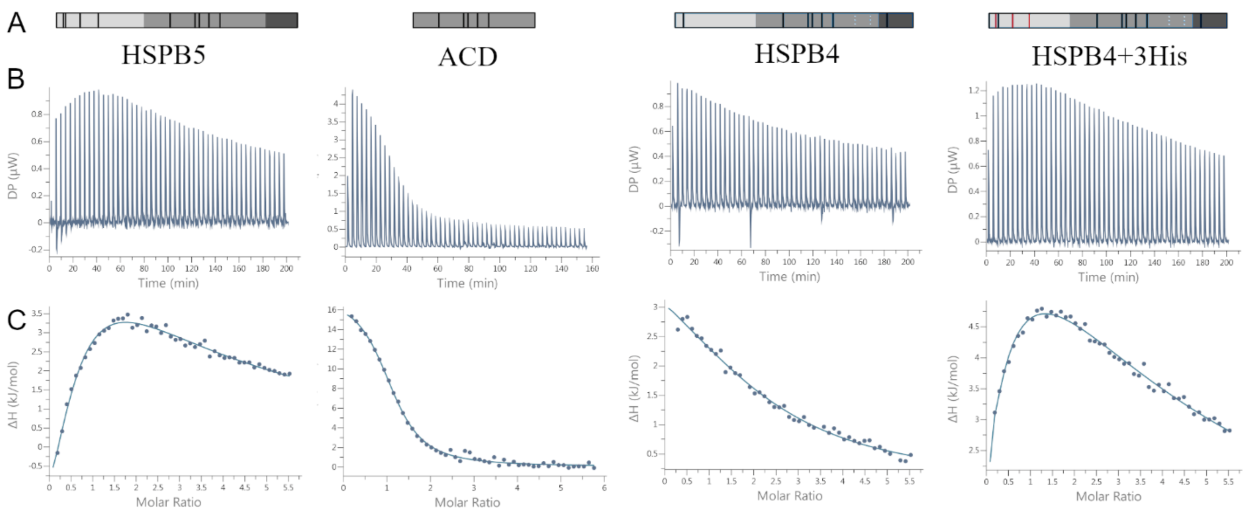

Figure S3. ITC profiles. A. Schematic representation of the domain's regions. B. ITC injection profiles. C. Isotherms with fitted curves (either 2 or 1 binding site).

	Protein	ΔH_1 (kJ/mol)	ΔG_1 (kJ/mol)	$-T\Delta S_1$ (kJ/mol)	ΔH_2 (kJ/mol)	ΔG_2 (kJ/mol)	$-T\Delta S_2$ (kJ/mol)	Red. Chi-Sqr. $(kJ/mol)^2$
1	HSPB5 pH 7.5	-173	-26.6	146	6.25	-20.5	-26.8	6.20×10^{-3}
2	HSPB5 pH 6.5	-261	-26.4	235	5.01	-19.8	-24.8	9.90×10^{-3}
3	HSPB4	0	0	0	5.1	-23.6	-28.7	5.50×10^{-3}
4	HSPB4-3His	-3.8	-26.5	-22.7	4.92	-19.5	-24.5	5.40×10^{-3}
5	ACD, pH 6.5	0	0	0	17.7	-29.3	-46.9	6.10×10^{-2}

Table S1 Fit parameters for ITC profiles.

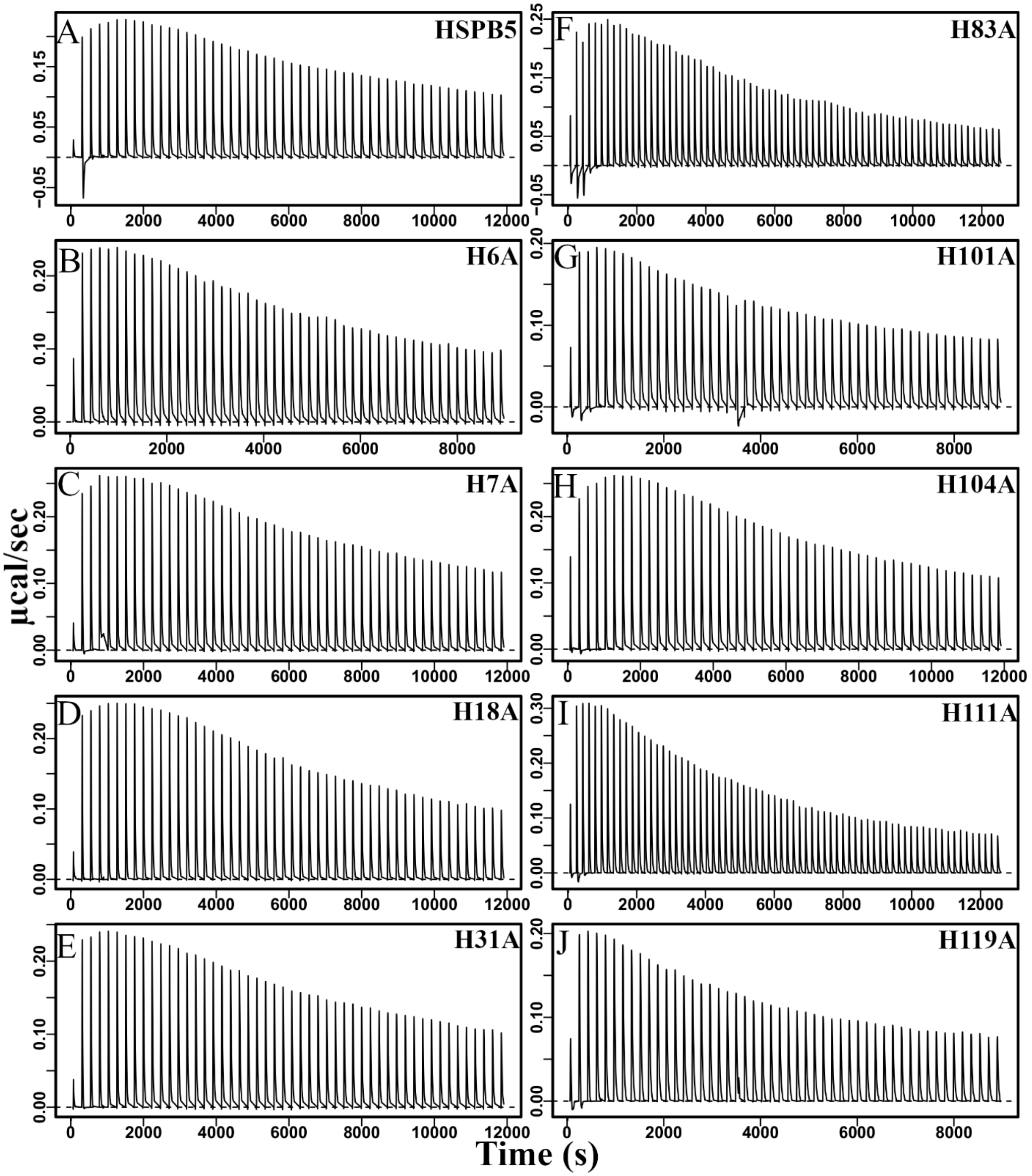

Figure S4 ITC injection profiles. Histidine-to-Alanine mutants of HSPB5.

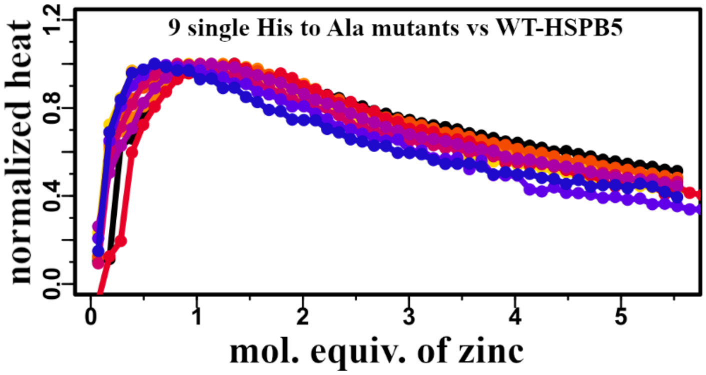

Figure S5 Normalized heat derived from the ITC experiment for His-to-Ala mutants of HSPB5. The overlay of the 9 single His to Ala mutants with WT HSPB5 shows that the biphasic pattern of the HSPB5 isotherm upon zinc binding is retained for every variant.

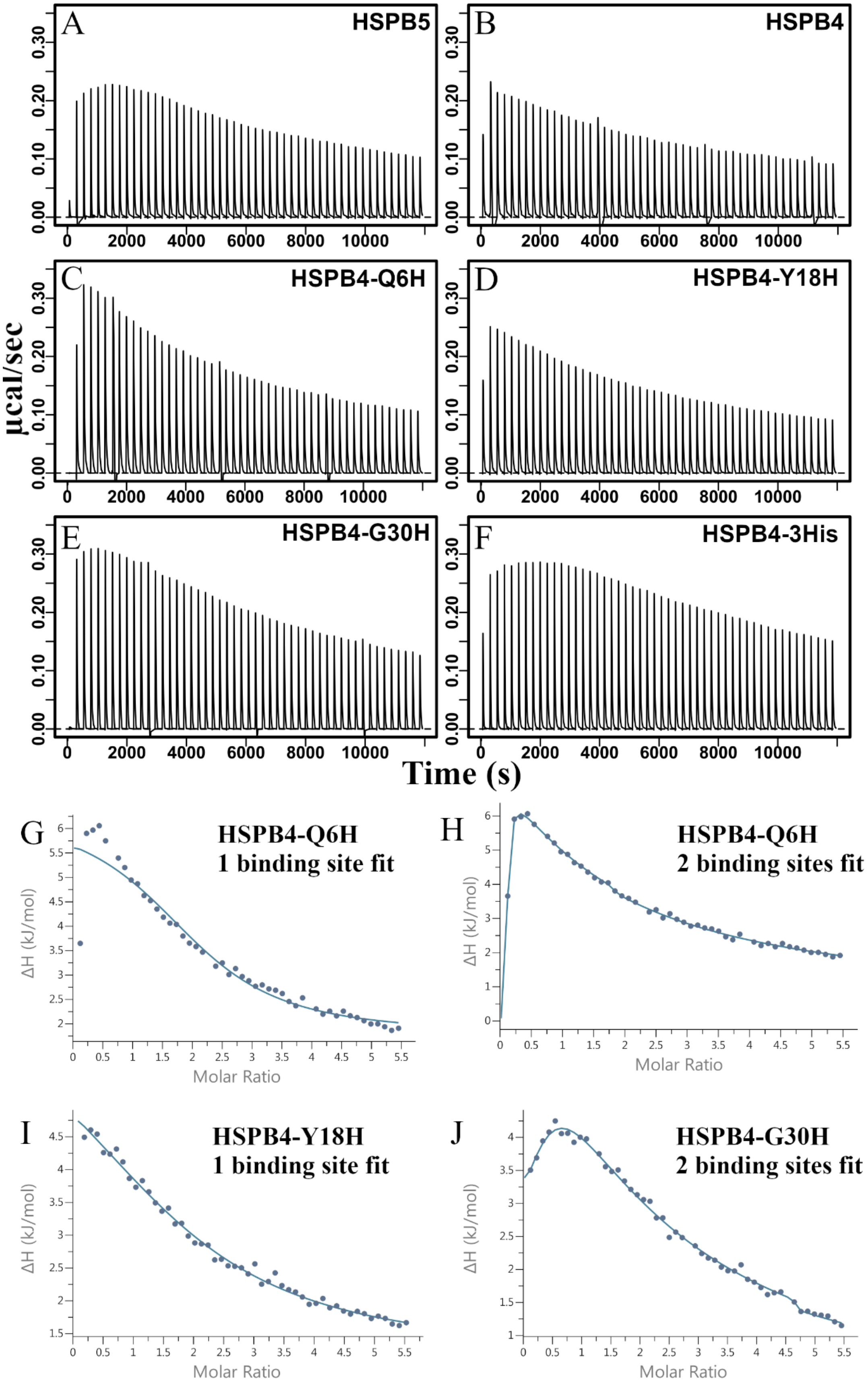
A
HSPB5
B
HSPB4
C
HSPB4-Q6H
D
HSPB4-Y18H
E
HSPB4-G30H
F
HSPB4-3His
µcal/sec
Time (s)
G
HSPB4-Q6H
1 binding site fit
H
HSPB4-Q6H
2 binding sites fit
I
HSPB4-Y18H
1 binding site fit
J
HSPB4-G30H
2 binding sites fit
ΔH (kJ/mol)
Molar Ratio

Figure S6 A-F ITC injection profiles. A. HSPB5, B. HSPB4, C. HSPB4-Q6H, D. HSPB4-Y18H, E. HSPB4-G30H, and F. HSPB4-3His in which all 3 NTR histidine installed. G-J Isotherms with fitted curves. G. HSPB4-Q6H, single transition fit does not recapitulate the data well. C. HSPB4-Q6H 2 transition fit, D. HSPB4-Y18H single transition, E. HSPB4-G30H 2 transitions fit.

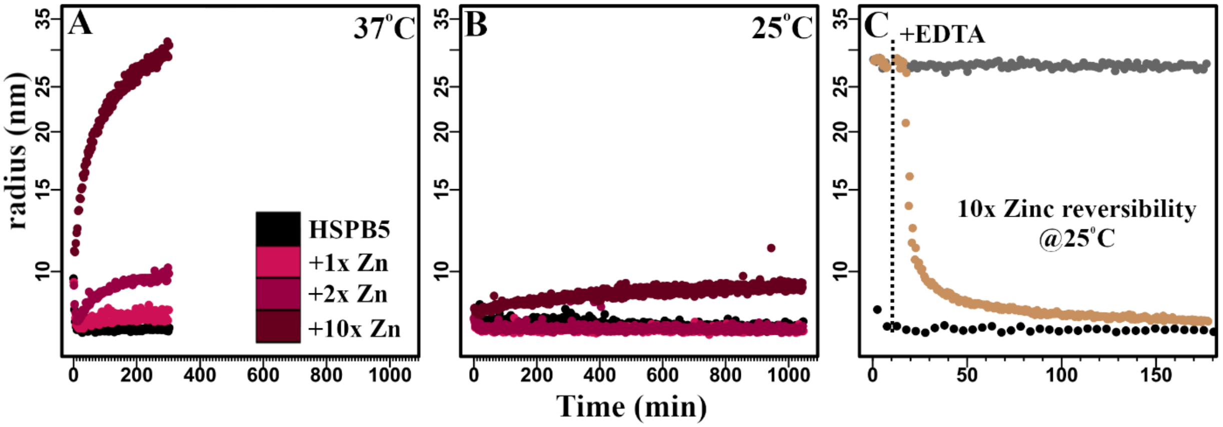

Figure S7 Effect of temperature on the zinc-induced change in HSPB5 size as measured by DLS. A. Change in size of HSPB5 at 37°C (no zinc (black), 1x zinc (magenta), 2x zinc (dark pink), 10x zinc (maroon)) (data from the main text figure 2). B. Change in size of HSPB5 at 25°C (no zinc (black), 1x zinc (magenta), 2x zinc (dark pink), 10x zinc (maroon)). Axes for Panels A and B are shown in the same range to allow direct comparison. The Y-axis is logarithmic to focus on smaller particles. C. The reversibility of formation of high-order species HSPB5 by addition of EDTA at 25°C. Oligo-oligomeric HSPB5 was generated in the presence of 10x zinc at 37°C. Samples were then placed in a plate reader at 25°C and the DLS signal was monitored at 25°C. Complete return to original size required >100 min. Apo-HSPB5 (black), preincubated HSPB5 with 10x zinc (oligo-oligomers) (grey) , preincubated HSPB5 with 10x zinc +EDTA added at the timepoint indicated by dotted line (beige). Experiments performed in duplicate.

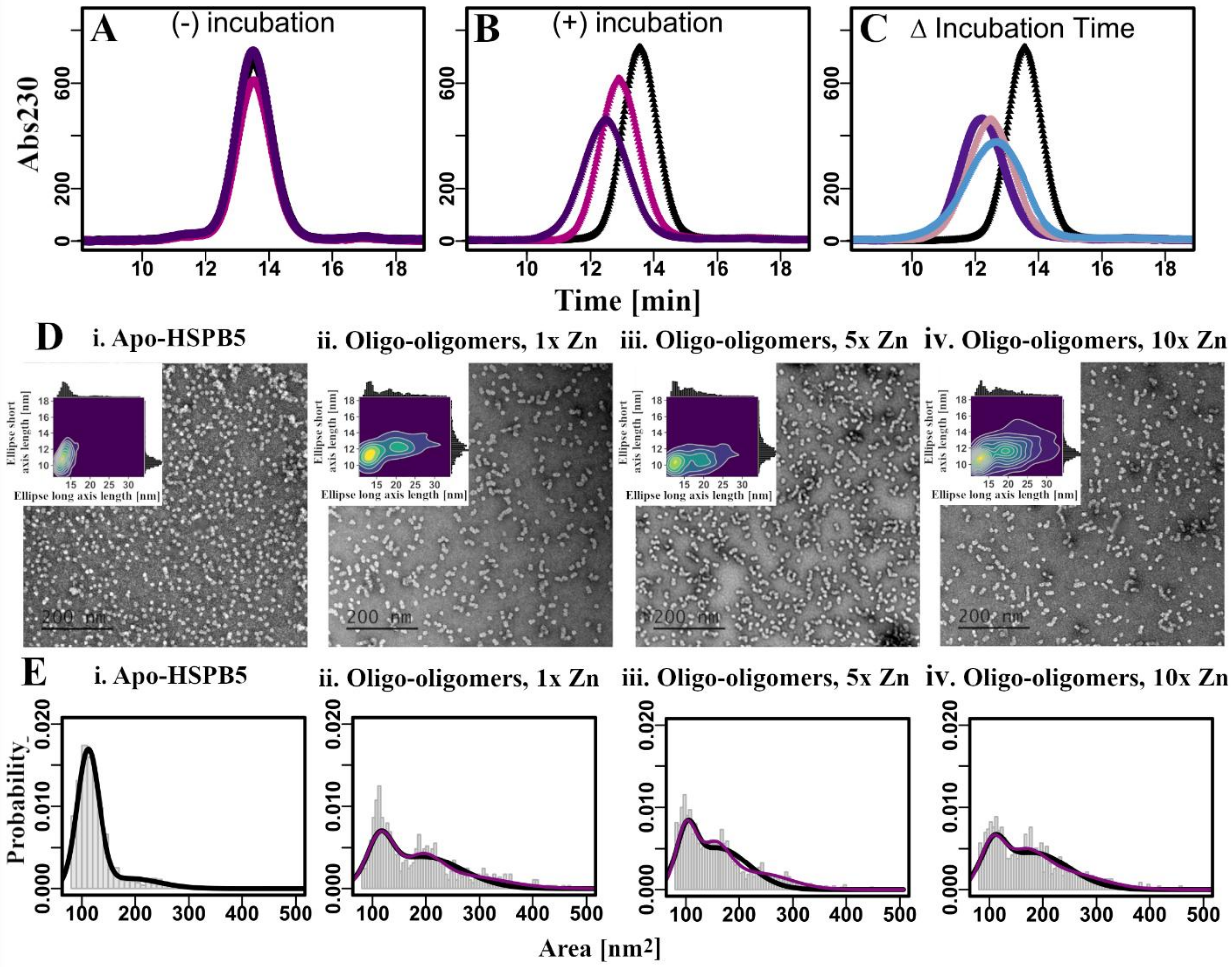

Figure S8 Conformational rearrangement of HSPB5 upon zinc binding. A. SEC performed at room temperature of HSPB5 with 0x (black), 1x (pink), or 2x (dark pink) of zinc on ice, without incubation at 37°C. B. HSPB5 with 0x (black), 1x (pink), or 2x (dark pink) zinc after 3h incubation at 37°C. C. SEC of HSPB5 with 2x zinc after different incubation time @37: 0h (black), 1h (blue), 3h (pink), 18h (purple). D. i-iv. TEM images of HSPB5 in the presence of zinc. i. Apo-HSPB5 (image reused from Fig. 2C.i), ii. Oligo-oligomers, 1x zinc, iii. Oligo-oligomers, 5x Zn., iv. Oligo-oligomers, 10x Zn (image reused from Fig. 2C.iii). Insets: EM images were processed using Fiji and ellipsoid was fitted into the particles. Plots show 2D density plots of fitted minimal short and long ellipsoid axis. Upon incubation with zinc there is an increase in size on long axis of the ellipsoid supporting formation of cross-oligomers interactions. E. Histograms of HSPB5 area distribution (grey bars) with fits to 2 (black line) or 3 (purple line) Gaussians, i. Apo-. ii. Oligo-oligomers, 1x zinc, iii. Oligo-oligomers, 5x Zn., iv. Oligo-oligomers, 10x Zn.

A

Species	# particles
Apo	998
Mono-oligomers, 10x Zinc	1636
Oligo-oligomers; 10xZinc + EDTA	996
Oligo-oligomers, 1x Zinc	578
Oligo-oligomers, 5x Zinc	1422
Oligo-oligomers, 10x Zinc	949

B

Species	% monomers (% AUC 1st Gauss)	% oligomers (AUC 2nd Gauss)
Apo	83.3	16.7
Mono-oligomers, 10x Zinc	71.2	28.8
Oligo-oligomers; 10xZinc + EDTA	87.0	13.0
Oligo-oligomers, 1x Zinc	27.5	72.5
Oligo-oligomers, 5x Zinc	24.0	76.0
Oligo-oligomers, 10x Zinc	22.0	78.0

C

Species	% monomers (% AUC 1st Gauss)	% dimers (AUC 2nd Gauss)	% oligomers (AUC 3nd Gauss)
Oligo-oligomers, 1x Zinc	41.5	26.4	32.1
Oligo-oligomers, 5x Zinc	33.6	36.8	29.6
Oligo-oligomers, 10x Zinc	29.2	32.2	38.6

Table S2 Summary of analysis of EM images. A. Number of particles used for analysis. B. Area under the curve for different species of HSPB5 fitted with 2 gaussians. C. Area under the curve for oligo-oligomers samples fitted with 3 Gaussians.

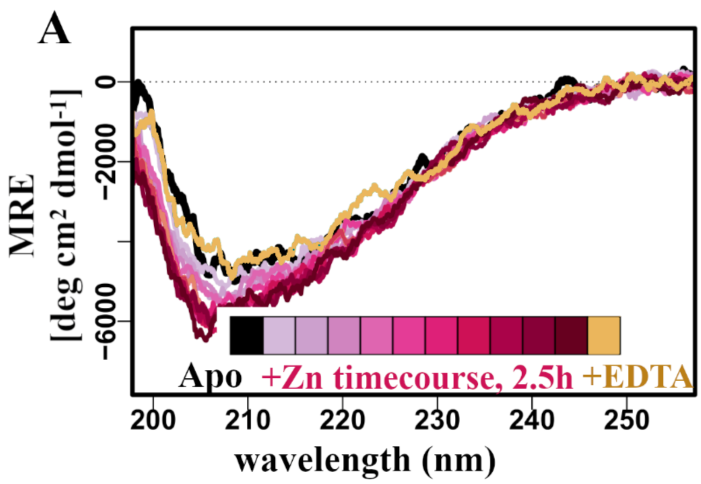

Figure S9 Conformational rearrangement of HSPB5 in the presence of zinc. A) CD spectra of 2.5 µM HSPB5 in the presence of 30x zinc collected in 15 min interval over 2.5h time period at 37°C. Spectrum of HSPB5 in the absence of zinc is in black, time course illustrating conformational rearrangement post-zinc addition is in magenta color gradient. Spectrum collected after addition of EDTA is shown in tan demonstrates reversibility, as it overlays with the black spectrum.

**Species**	**k_{ex} (1/s)**			**Average k_{ex} (1/s)**	
	#1	#2	#3		
No zinc	$3.5x10^{-4}$	$3.0x10^{-4}$	$2.4x10^{-4}$	$2.9±(0.55)x10^{-4}$	1 subunit: 1 hour
0.2x zinc	$7.0x10^{-5}$	$6.7x10^{-5}$	$7.4x10^{-5}$	$7.0±(0.35)x10^{-5}$	1 subunit: 4h
2x zinc	$8.8x10^{-6}$	$5.5x0^{-6}$	$3.3x10^{-6}$	$5.9±(2.7)x10^{-6}$	1 subunit: 50h

Table S3 Subunit exchange fit parameters.

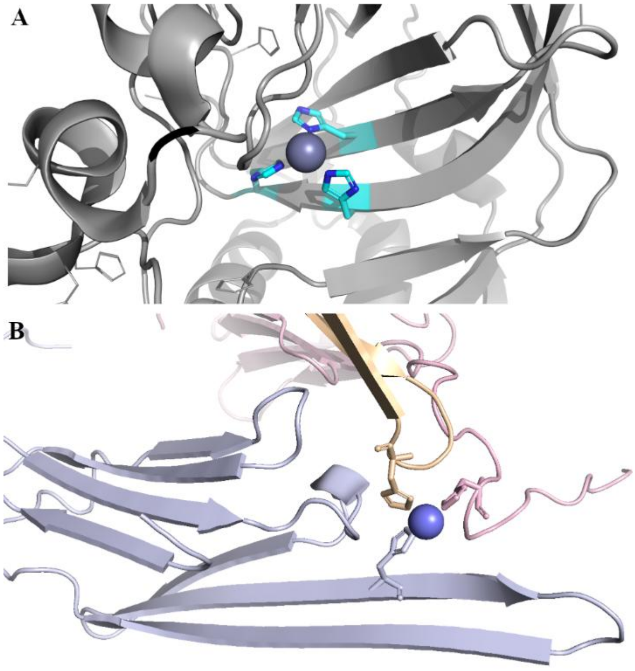

Figure S10 PDB structures of zinc-binding proteins coordinated by three histidine residues. A. α-Carbonic anhydrase from Persephonella marina EX-H1 (PDB: 6IM1). B. ACD of HSPB4, which completes the coordinating sphere using histidines from neighboring crystallographic subunits. The subunits do not form the canonical ACD dimer but are positioned in neighboring subunits, where they interact through the loops. In both structures, zinc (dark blue sphere) is coordinated by histidine residues shown as sticks. For clarity, the three subunits of the ACD are shown in different colors.

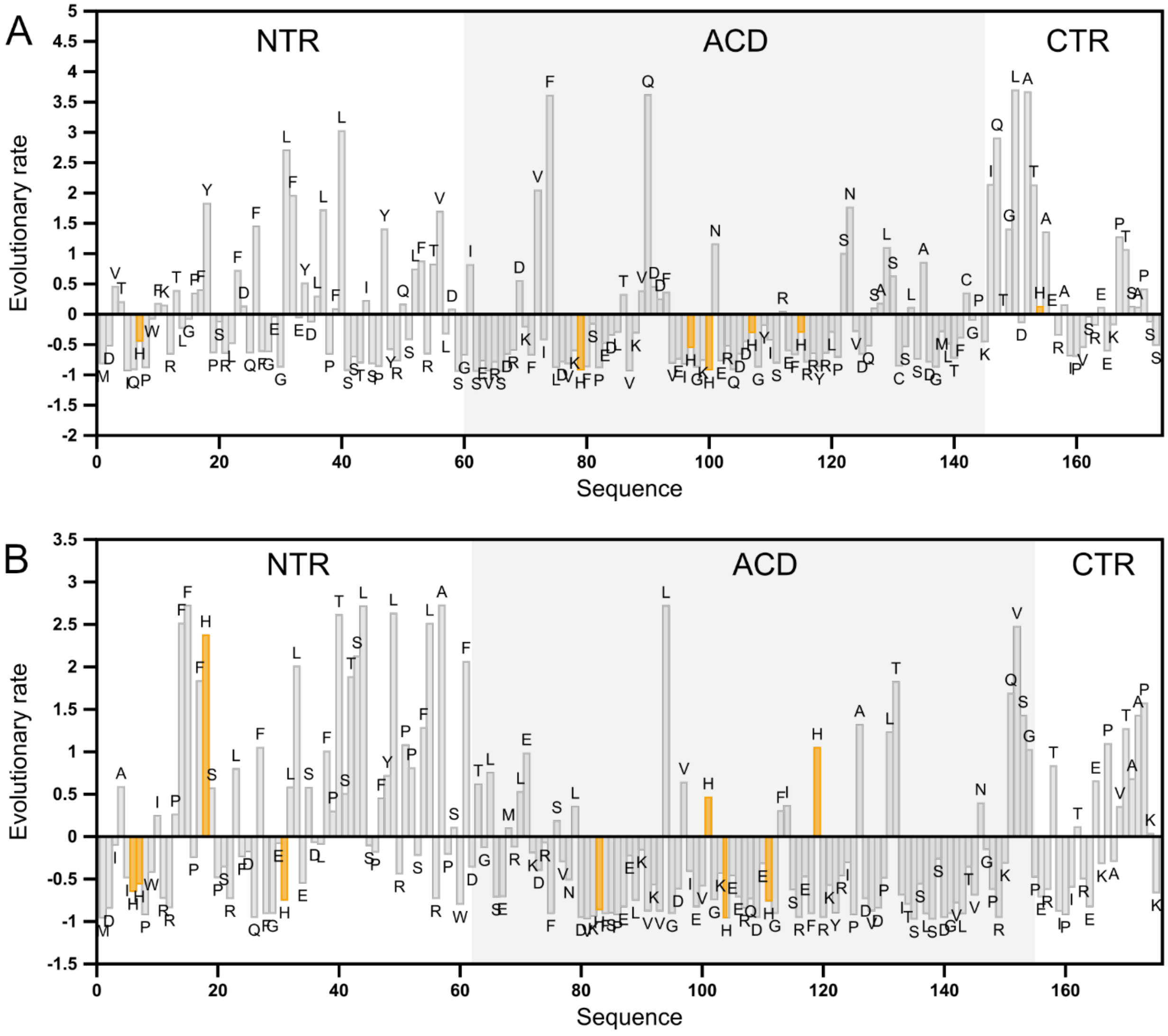

Figure S11 Normalized evolutionary rate profile of human A. HSPB4 B. HSPB5. Each bar represents the site-specific evolutionary rate estimated from a multiple sequence alignment of vertebrate orthologs. Histidine residues are shown in orange. The ACD boundaries are shaded in light grey.

sample#	mg/L	mg/L	mg/L	mg/L	mg/L	mg/L	mg/L	mg/L	mg/L	mg/L	mg/L
	Ag3382	Al3092	As1890	B_2497	Ba4554	Ca3158	Cd2288	Cr2677	Cu3247	Fe2395	K_7698
Sample-1	-0.0052	0.0267	0.0012	-0.0028	0	-0.0089	0	-0.0004	0.0005	0.0045	0.1262
Sample-2	-0.0046	0.0032	0.0005	0.0062	-0.0006	-0.0116	0	0.0028	0.0002	0.0042	-0.0138
Sample-3	0.0027	0.0048	0.0025	0.0115	0.0002	0.1051	0.0001	0.0001	-0.0002	0.0087	8.765
Sample-4	-0.0025	0.0108	0.0094	0.0135	-0.0002	0.4816	-0.0001	0.0015	-0.0024	0.0033	11.34
Sample-5	-0.0007	0.0101	0.0066	0.0109	-0.0003	0.0353	0.0001	0.0002	-0.0025	0.0044	6.21
Sample-6	0.0028	0.0107	0.0069	0.0085	-0.0002	-0.009	0.0002	0.0031	-0.0036	0.0032	6.027
Sample-7	-0.0041	0.0088	0.006	0.0083	-0.0004	-0.0058	0	0.0015	-0.0003	0.0049	5.947
Sample-8	-0.0039	0.0139	0.0067	0.0091	-0.0004	-0.0078	-0.0001	0.0016	0.0066	0.0052	5.742
Sample-9	-0.002	0.0081	-0.0006	0.0002	-0.0005	-0.0063	-0.0001	0.0024	0.0003	0.0026	0.2822
QC run	4.899	25.14	5.019	5.112	4.953	24.43	5.018	5.134	5.084	5.067	49.16
QC rtrue	5.000	5.000	5.000	5.000	5.000	25.000	5.000	5.000	5.000	5.000	50.000

sample#	mg/L	mg/L	mg/L	mg/L	mg/L	mg/L	mg/L	mg/L	mg/L	mg/L	mg/L
	Mg2852	Mn2576	Mo2020	Na5895	Ni2316	P_2149	Pb2203	S_1820	Se1960	Si2881	Zn2138
Sample-1	-0.0034	-0.0004	0	-0.0413	0.0001	-0.0064	0.0006	-0.0088	0.0026	-0.1235	0.0001
Sample-2	-0.0029	-0.0003	0.0026	-0.0077	0.0003	-0.0002	0.0009	0.0015	0.0015	-0.1024	0.004
Sample-3	0.0007	-0.0003	0.002	714.8	0.0007	0.0057	0.0009	716.1	0.0003	0.1252	0.0009
Sample-4	0.0356	0.0012	0.0005	518.7	0.0003	0.3946	0.0003	1515	0.006	0.091	0.0019
Sample-5	0.0095	-0.0003	0.0013	512.8	0.0002	0.2671	0.0014	1252	0.0046	0.036	0.0058
Sample-6	-0.0048	-0.0002	0.0012	494.4	0.0006	0.2654	0.0011	1290	0.0022	0.0269	0.0044
Sample-7	-0.0047	-0.0006	0.0006	490.4	0.0002	0.7211	0.0002	1316	0.0049	0.0124	0.0089
Sample-8	-0.0054	-0.0006	0.0008	481.5	0	3.951	0.0002	1338	0.0051	0.0261	0.0047
Sample-9	-0.0039	-0.0006	0.0002	2.972	-0.0002	0.0016	0.0007	0.9861	0.0004	-0.0963	0.0013
QC run	4.899	4.915	4.918	10.06	4.825	9.766	5.049	4.898	5.086	5.056+	5.067
QC rtrue	5.000	5.000	5.000	10.000	5.000	10.000	5.000	5.000	5.000	5.000	5.000

Sample description
1. MiliQ water
2. Optima Water
3. 25mM MOPS, 150mM NaCl, pH 7.5, "normal" buffer made with MiliQ water
4. 50mM HEPES, 100mM NaCl,pH 7.4 made with optima water, before chelexing
5. #4 +chelexed for 3h @37C (1mg resin to 10ml of buffer)+ filtered through syringe
6. #4 +chelexed for 3h @37C (1mg resin to 10ml of buffer), unfiltered
7. 3uM HSPB5 buffer exchanged to buffer # 5
8. 3.75 uM HSPB5 in PBS (purification buffer) diluted with buffer #5
9. FluoZin-3 400nM

Figure S12 Metal concentrations in the buffer and sample were quantified using inductively coupled plasma mass spectrometry.

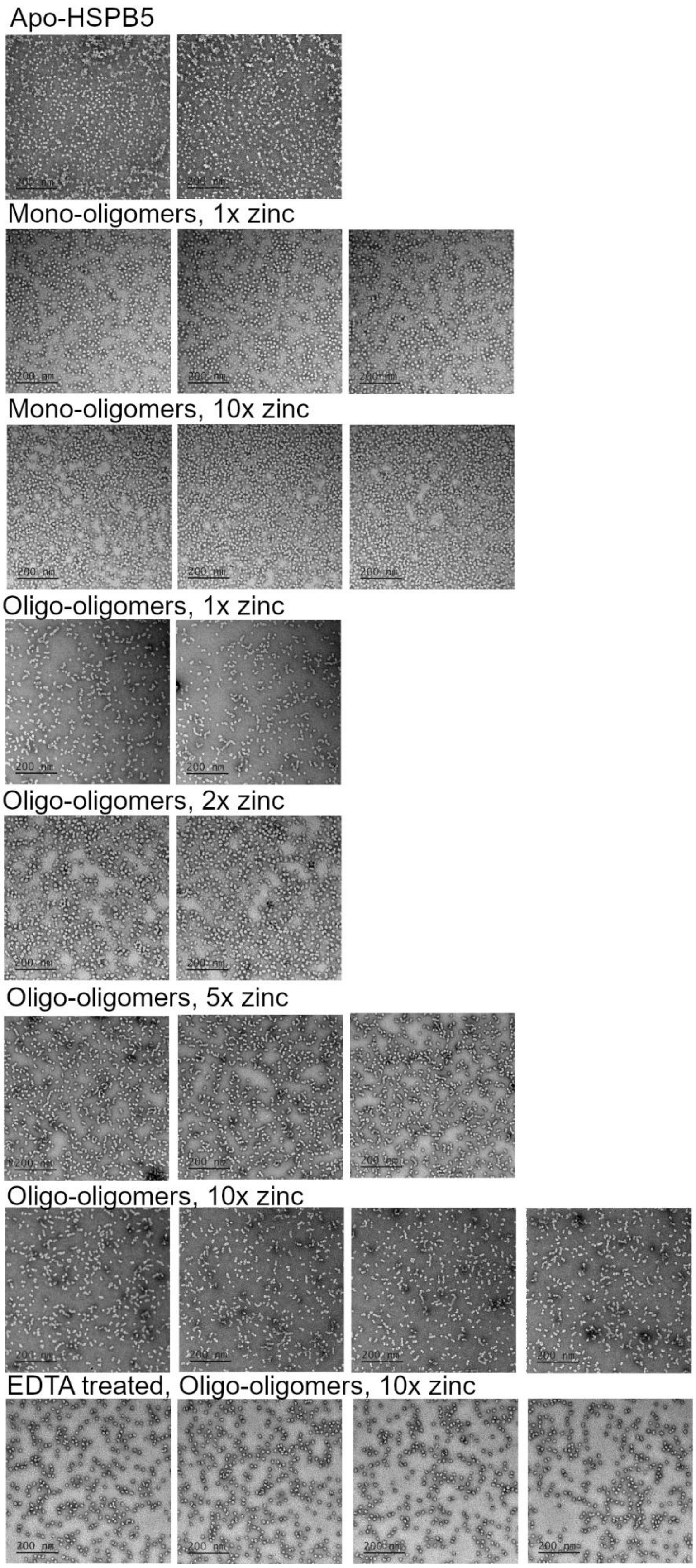
Apo-HSPB5
Mono-oligomers, 1x zinc
Mono-oligomers, 10x zinc
Oligo-oligomers, 1x zinc
Oligo-oligomers, 2x zinc
Oligo-oligomers, 5x zinc
Oligo-oligomers, 10x zinc
EDTA treated, Oligo-oligomers, 10x zinc
200 nm

Figure S13 TEM micrographs of HSPB5 species. One image of each series is reused from Figure 2C and/or Figure S8.

References

1. Delbecq, S. P., Jehle, S., and Klevit, R. (2012) Binding determinants of the small heat shock protein, αB-crystallin: recognition of the "IxI" motif. *EMBO J.* **31**, 4587–4594
2. Woods, C. N., Ulmer, L. D., Guttman, M., Bush, M. F., and Klevit, R. E. (2023) Disordered region encodes α-crystallin chaperone activity toward lens client γD-crystallin. *Proc Natl Acad Sci U S A*. **120**, e2213765120
3. Jakob, U., Eser, M., and Bardwell, J. C. (2000) Redox switch of hsp33 has a novel zinc-binding motif. *J Biol Chem*. **275**, 38302–38310
4. Elzhov, T. V., Mullen, K. M., Spiess, A.-N., and Bolker, B. (2016) minpack.lm: R Interface to the Levenberf-Marquardt Nonlinear Least-Squares Algorithm Found in MINPACK, Plus Support for Bounds
5. Marszałek, I., Krężel, A., Goch, W., Zhukov, I., Paczkowska, I., and Bal, W. (2016) Revised stability constant, spectroscopic properties and binding mode of Zn(II) to FluoZin-3, the most common zinc probe in life sciences. *Journal of Inorganic Biochemistry*. **161**, 107–114
6. Scheuermann, T. H., and Brautigam, C. A. (2015) High-precision, automated integration of multiple isothermal titration calorimetric thermograms: New features of NITPIC. *Methods*. **76**, 87–98
7. Clouser, A. F., Baughman, H. E., Basanta, B., Guttman, M., Nath, A., and Klevit, R. E. (2019) Interplay of disordered and ordered regions of a human small heat shock protein yields an ensemble of "quasi-ordered" states. *Elife*. **8**, e50259
8. Tarutani, A., and Hasegawa, M. (2021) Electron Microscopic Analysis of α-Synuclein Fibrils. *Methods Mol. Biol*. **2322**, 17-25,
9. Schneider, C. A., Rasband, W. S., and Eliceiri, K. W. (2012) NIH Image to ImageJ: 25 years of image analysis. *Nat Methods*. **9**, 671–675
10. Okonechnikov, K., Golosova, O., Fursov, M., and UGENE team (2012) Unipro UGENE: a unified bioinformatics toolkit. *Bioinformatics*. **28**, 1166–1167
11. Pupko, T., Bell, R. E., Mayrose, I., Glaser, F., and Ben-Tal, N. (2002) Rate4Site: an algorithmic tool for the identification of functional regions in proteins by surface mapping of evolutionary determinants within their homologues. *Bioinformatics*. **18 Suppl 1**, S71-77
12. Jones, D. T., Taylor, W. R., and Thornton, J. M. (1992) The rapid generation of mutation data matrices from protein sequences. *Comput Appl Biosci*. **8**, 275–282
13. Saitou, N., and Nei, M. (1987) The neighbor-joining method: a new method for reconstructing phylogenetic trees. *Mol Biol Evol*. **4**, 406–425
14. Rajagopal, P. (2015) A conserved histidine modulates HSPB5 structure to trigger chaperone activity in response to stress-related acidosis. *Elife*. 10.7554/eLife.07304.
